# Case Report: *Listeria monocytogenes meningitis* complicated by an acute exacerbation of chronic obstructive pulmonary disease: the key diagnostic role of metagenomic high-throughput sequencing

**DOI:** 10.3389/fmedt.2026.1801483

**Published:** 2026-06-10

**Authors:** Yiwei Chen, Dacheng Tian, Yixuan Bai, Jinhui Xu, Shuaichun Liu, Yanwen Wang, Xingfang Li

**Affiliations:** 1 School of Integrated Traditional Chinese and Western Medicine, Gansu University of Chinese Medicine, Lanzhou, Gansu, China; 2 Department of Pulmonology, Gansu Provincial Hospital of Traditional Chinese Medicine, Lanzhou, Gansu, China; 3 Respiratory Department, Wuwei Liangzhou Hospital, Wuwei, Gansu, China

**Keywords:** case report, chronic obstructive pulmonary disease (COPD), listeria monocytogenes, meningitis, metagenomic next-generation sequencing (mNGS)

## Abstract

**Background:**

*Listeria monocytogenes* is an opportunistic foodborne pathogen that causes severe invasive infections, such as meningitis, primarily in immunocompromised individuals, the elderly, and pregnant women. Diagnosis is often challenging due to nonspecific early symptoms.

**Case description:**

A 67-year-old male with a history of chronic obstructive pulmonary disease (COPD) presented with a 4-day history of persistent high-grade fever and altered mental status. Initial empirical antibiotic therapy (meropenem) proved ineffective.Metagenomic next-generation sequencing (mNGS) of cerebrospinal fluid (CSF) definitively identified *L.monocytogenes*. The patient was diagnosed with “Listeria monocytogenes meningitis complicated by an acute exacerbation of chronic obstructive pulmonary disease”. Patients with pathogenic bacterial infections completed a 21-day course of ampicillin and sulbactam sodium and a 14-day course of gentamicin, resulting in a rapid improvement in clinical symptoms and biochemical parameters.

**Conclusion:**

This case underscores the critical role of mNGS in the aetiological diagnosis of central nervous system infections, especially when conventional methods are inconclusive. It highlights the need for a high index of suspicion for listeriosis in elderly patients with comorbidities presenting with unexplained fever and neurological decline.

## 1 Introduction

*Listeria monocytogenes*, a Gram-positive, facultative intracellular bacterium, is a significant cause of foodborne illness with a high mortality rate in invasive disease. It is ubiquitous in the environment and commonly associated with contaminated ready-to-eat foods, unpasteurized dairy products, and processed meats. While immunocompetent individuals may experience only mild febrile gastroenteritis, *L. monocytogenes* can cause severe, life-threatening infections including sepsis, meningitis, and encephalitis in vulnerable populations such as neonates, pregnant women, the elderly, and immunocompromised hosts. Transmission primarily occurs via the fecal-oral route (1).

The pathogenesis of central nervous system invasion involves complex interactions between bacterial virulence factors and the host immune response. Key virulence proteins, including internalin A (InlA) and B (InlB), facilitate bacterial crossing of the intestinal and blood-brain barriers by binding to host receptors like E-cadherin and Met, respectively (2–4). Following phagocytosis, listeriolysin O (LLO) and phospholipases mediate the bacteria's escape from phagosomes, thereby enabling them to replicate within the host cell cytoplasm and subsequently spread between cells (5). The resulting infection triggers a vigorous inflammatory cascade, leading to disruption of the blood-brain barrier and neuronal damage.

Conventional pathogen detection methods, including culture, staining and targeted PCR, are limited by low positivity rates, long turnaround times and narrow detection ranges. Metagenomic next-generation sequencing (mNGS) (6) is a hypothesis-free, unbiased and highly sensitive tool capable of simultaneously detecting multiple pathogens directly from clinical samples. This technology is of critical value for culture-negative or clinically unsuspected pathogen infections.

Here, we present a case of severe *L. monocytogenes* meningitis in an elderly patient with underlying COPD, where mNGS was instrumental in establishing a timely diagnosis after initial diagnostic uncertainty, guiding successful targeted therapy.

## 2 Case presentation

### 2.1 History and initial presentation

A 67-year-old male presented on September 24, 2025, with a chief complaint of persistent fever and progressive lethargy for four days. The illness began on September 20 after a cold exposure, with an initial moderate fever (up to 38.9 °C), chills, generalized weakness, dizziness, and headache. He was initially admitted to a local hospital. Laboratory tests revealed leukocytosis (white blood cell count, WBC 13.78 × 10⁹/L)with neutrophil percentage82.90%,lymphocyte percentage (10.50%), absolute neutrophil count (ANC)11.42 × 10⁹/L, and elevated C-reactive protein (CRP, 31.45 mg/L). Despite four days of empirical antimicrobial and supportive therapy (detailed agents not specified by referring institution), his condition deteriorated with a rising fever (up to 39.8 °C) and worsening mentation.

His past medical history was significant for pulmonary tuberculosis (diagnosed and treated 50 years prior), chronic obstructive pulmonary disease (COPD) for 7-8 years with poor medication adherence, and a history of radiofrequency ablation five years ago, after which he was maintained on metoprolol sustained-release tablets (47.5 mg daily). The patient had no history of corticosteroid or immunosuppressant use. He reported no recent travel or sick contacts. Upon further questioning after diagnosis, he admitted to frequent consumption of long-term refrigerated meats and leftovers in the preceding weeks, with occasional mild, self-limited gastrointestinal discomfort.

### 2.2 Clinical findings on admission

On admission to our institution, the patient was febrile (39.4 °C), tachycardic (102 bpm), and tachypneic (26 breaths/min), with a blood pressure of 124/65 mmHg. He appeared lethargic. According to the family, the patient had experienced intermittent coughing and sputum production for approximately one week; the sputum was copious and yellowish-white in colour, chest tightness and shortness of breath, with wheezing and a feeling of suffocation during daily activities and even at rest. Walking and daily activities were significantly restricted, with symptoms worsening at night. The patient also complained of fatigue, poor mental state, loss of appetite and poor sleep. Based on the symptoms described by the family, the VAS score was 8 and the CAT score was 21.

Neurological examination revealed lethargy and disorientation. He was responsive to verbal stimuli but gave incoherent answers. The Glasgow Coma Scale (GCS) score was 14. No focal motor or sensory deficits, meningeal irritation signs, or pathological reflexes were identified. Pupils were equal, round, and reactive to light. Cardiopulmonary examination revealed barrel chest, bilateral vocal fremitus was diminished, hyperresonance on percussion, prolonged expiratory phase, bilateral lower lobe crackles and significant pitting edema of the lower extremities. Dorsalis pedis pulses were weakly palpable.

### 2.3 Diagnostic investigations

#### 2.3.1 Imaging

Chest computed tomography (CT) showed bilateral pulmonary interstitial changes, emphysema, bullae, and infectious foci in both lower lobes, combined with clinical symptoms and physical examination findings, this is consistent with acute exacerbation of COPD. Cranial CT revealed only age-related cerebral atrophy and subcortical arteriosclerotic encephalopathy.

#### 2.3.2 Laboratory studies

24 September 2025: Laboratory tests upon admission confirmed persistent and significant leukocytosis (WBC 15.84 × 10⁹/L), neutrophilia (neutrophil percentage 86.1%, absolute neutrophil count 13.65 × 10⁹/L), a reduced lymphocyte percentage (5.56%) and a sharp rise in C-reactive protein to 95.79 mg/L. Testing for novel coronavirus nucleic acid and respiratory pathogens (influenza A virus, influenza B virus, respiratory syncytial virus, human rhinovirus, *Mycoplasma pneumoniae*) was negative in all cases Table 1.

**Table 1:** Changes in infection indicators from admission to discharge.

Item	9.23	9.24	9.25	9.26	9.27	9.28	9.29	9.30	10.01	10.08	10.14
WBC ( × 10^9^/L)	13.78	15.84	11.37	9.79	9.89	11.71	12.39	10.43	8.54	6.64	7.02
neutrophils (%)	82.90	86.10	80.00	75.00	71.20	71.50	74.00	68.00	71.40	64.10	62.20
lymphocytes (%)	10.50	5.60	8.40	14.40	18.30	16.90	12.80	17.90	12.80	21.20	22.50
ANC ( × 10^9^/L)	11.42	13.65	9.09	7.34	7.04	8.38	9.18	7.08	6.10	4.25	4.37
CRP (mg/L)	31.45	95.79	71.86	48.14	24.80	18.22	19.71	18.84	14.67	＜6	＜6
PCT(ng/mL)	**/**	1.92	1.28	0.85	0.57	0.38	0.25	0.17	0.11	0.08	0.05

A video bronchoscopy was performed on 25 September 2025. Under bronchoscopy, a small amount of carbon-like pigment deposits was observed at the opening of the basal segment of the left lower lobe; no neoplasms were observed. A small amount of thin, white secretions was observed within the lumen of the right bronchus and was aspirated. No significant abnormalities were noted in the glottis, trachea or carina. Bronchoalveolar lavage fluid and brush specimens were collected from the basal segment of the left lower lobe, and the basal segments of the right middle and lower lobes, and sent for examination. A lumbar puncture was performed at 17:00 on the same day; the initial puncture pressure was 220 mmH₂O, and 28 mL of cerebrospinal fluid was slowly aspirated. Routine, biochemical and general bacterial smear examinations were performed, and the specimen was sent externally for metagenomic sequencing (mNGS). Analysis of the cerebrospinal fluid revealed a protein-cell dissociation: total protein was significantly elevated (2,779 mg/L), glucose was reduced (0.78 mmol/L), albumin was 1,578 mg/L, white blood cell count was 120/mL, with neutrophils accounting for 53%, lymphocytes 28%, and monocytes 19%. After 5 days of incubation, a few colonies of Listeria monocytogenes were recovered from the cerebrospinal fluid culture. (consistent with the mNGS results); venous blood cultures were negative.

#### 2.3.3 Metagenomic next-generation sequencing

Given the patient's persistent fever and persistently elevated infection markers, the diagnosis reached an impasse. Consequently, on 25 September 2025 at 17:00, cerebrospinal fluid (sample number: MBX267095) and bronchoalveolar lavage fluid (sample number: MBX268118) were collected and sent to Beijing GensKey Medical Testing Laboratory for mNGS testing. (Nucleic acid extraction was performed using the following kit: Model No. 2005-01, Lot No. MB2504001,DNA/RNA Library Preparation Kit Model: 2102 Lot Number: R062503010)The laboratory utilised a commercial pathogen DNA/RNA extraction kit for nucleic acid extraction and performed human host nucleic acid removal to enhance pathogen detection sensitivity. A sequencing library was constructed using a certified library preparation kit, and sequencing was performed on the Illumina NovaSeq 6000 platform using the ‘pair-ended 150 bp (PE150) mode’. Rigorous quality control is applied to the raw sequencing data. High-quality sequences are aligned against the human reference genome hg38 using Bowtie2 software to exclude host sequences. The remaining non-host sequences are analysed against a standardised microbial database (NCBI RefSeq + GensKey Medical's proprietary validation library) comprising bacteria, fungi, viruses and parasites. Species identification utilises high-precision alignment algorithms, with a sequence similarity of ≥97% at the species level deemed a valid match. A pathogen is confirmed when a unique, specific sequence is detected, parallel negative controls are negative, and the result is consistent with clinical manifestations; contamination interference is excluded through the laboratory's dynamic background microbial database and strict negative controls. (The relevant data is sourced from KingMed's testing platforms: https://www.genskey.com/).

On 27 September 2025, RNA analysis identified 602 Listeria spp. sequences, of which 310 were unique sequences specifically mapped to Listeria monocytogenes; DNA analysis identified 42,360 Listeria spp. sequences, of which 33,213 were unique sequences specifically mapped to Listeria monocytogenes. These results provide a definitive microbiological diagnosis for clinical management. No significant pathogens were detected in bronchoalveolar lavage fluid via mNGS testing. (The sequencing data has been published on NCBI under accession number PRJNA1450280) Tables 2, 3.

**Table 2:** mNGS DNA detection results of cerebrospinal fluid (CSF).

Type	Species name	Sequence counts	Relative abundance(%)	Species name	Sequence counts
G^+^	*Listeria*	42,360	98.26	*Listeria monocytogenes*	33,213

**Table 3:** mNGS RNA detection results of cerebrospinal fluid (CSF).

Type	Species name	Sequence counts	Relative abundance(%)	Species name	Sequence counts
G^+^	*Listeria*	602	3.365	*Listeria monocytogenes*	310

### 2.4 Treatment and clinical course

Initial empirical therapy for healthcare-associated meningitis and pulmonary infection was started with intravenous meropenem (2 g q8 h) and ampicillin/sulbactam (3 g q6 h). Given the patient's marked pitting oedema in both lower limbs and faint pulsations in the dorsalis pedis arteries, combined with a history of radiofrequency ablation suggesting new-onset heart failure and peripheral circulatory impairment, mannitol was administered to control intracranial pressure, diuretics were prescribed to manage heart failure, and dexamethasone was administered to reduce cerebral oedema through its anti-inflammatory effects. Additionally, symptomatic treatment and nutritional support were provided based on the results of relevant examinations and laboratory tests.

On the fourth day of admission, the patient was diagnosed with *Listeria monocytogenes* infection via metagenomic next-generation sequencing (mNGS). The antimicrobial treatment regimen was optimised: high-dose intravenous ampicillin/sulbactam (3 g q6 h) was continued, supplemented with gentamicin (5 mg/kg daily) for synergistic activity, while meropenem was discontinued. Supportive care was continued.

**Figure 1:**
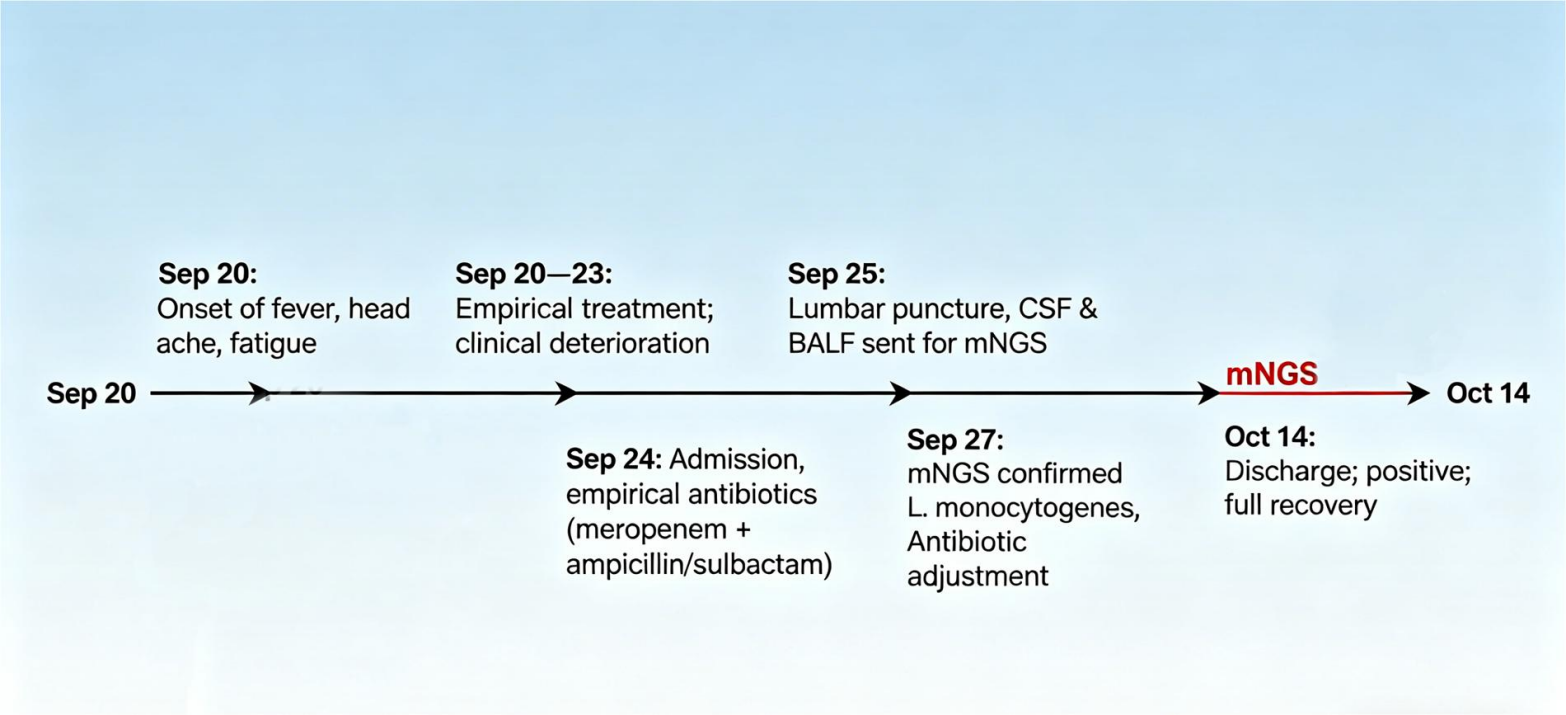
Timeline of diagnosis, mNGS testing and treatment.

The response was rapid. Within 24 h of targeted therapy, the patient's fever began to subside and mentation improved. Inflammatory markers trended down promptly (CRP decreased from 71.86 mg/L to 24.80 mg/L within one day). He completed a 21-day course of intravenous ampicillin/sulbactam and a 14-day course of gentamicin. On discharge, the patient was afebrile, alert and oriented, and able to get out of bed and move about. All laboratory parameters had returned to normal. No neurological sequelae, such as hearing loss or cranial nerve dysfunction, were observed at discharge or during the short-term follow-up period (Figure 1).

## 3 Discussion

Firstly, diagnosing *Listeria* infection in elderly patients with multiple comorbiditie presents certain challenges, as the symptoms may be non-specific and overlap with those of other acute conditions. This can lead to delayed initial diagnosis and negative results in routine cultures, resulting in a delayed diagnosis. Consequently, patients may receive standard symptomatic treatment, yet their condition may worsen.

Secondly, mNGS serves as a powerful diagnostic tool for identifying the cause of severe central nervous system infections of unknown origin. It enables rapid, unbiased and sensitive pathogen identification, without being limited by culture sensitivity. In this case, mNGS played a decisive role in the accurate diagnosis and timely adjustment of the treatment regimen. Compared with PCR-based methods (such as FilmArray and 16S rRNA PCR), mNGS, although requiring a longer turnaround time and incurring higher costs, provides unbiased results and covers a broader spectrum of pathogens. This technology is particularly valuable when both routine and targeted tests yield negative results.

Thirdly, with regard to treatment, high-dose ampicillin combined with gentamicin remains the gold standard for the treatment of *Listeria monocytogenes meningitis* (7, 8).Although meropenem is not a first-line agent, there was initially a lack of evidence for clinically targeted antimicrobial therapy. Given the drug's broad *in vitro* activity against both Gram-positive and Gram-negative pathogens (9), it was used to provide partial empirical coverage. Following confirmation of the diagnosis, treatment was switched to a regimen based on guidelines and expert consensus (10) and patients recovered rapidly, validating the efficacy of this treatment approach.

## 4 Conclusion

*Listeria monocytogenes meningitis* remains a severe infection with significant mortality, especially in vulnerable populations. High clinical vigilance is essential in elderly patients with comorbidities who present with unexplained fever and deteriorating neurological function. When conventional diagnostic methods fail to identify the causative agent, mNGS technology enables rapid and accurate aetiological diagnosis and guides life-saving targeted treatment. In complex cases of central nervous system infection, early mNGS testing should be considered to improve patient prognosis.

## Data Availability

The datasets presented in this study can be found in online repositories. The names of the repository/repositories and accession number(s) can be found in the article/Supplementary Material.
